# Collecting Doctor’s Fees

**Published:** 1887-02

**Authors:** 


### COLLECTING DOCTOR'S FEES.

The following remarks by a Country Doctor (which we find in the *Medical Age*) will, we think, meet the indorsement of all sensible men in the profession:

"My young friends, did it ever occur to you that a business man who is always prompt to meet his own obligations is generally equally so in collecting bills that may be due him? Pay your own bills promptly, or, if possible, make no bills, but pay for what you get or do without. You may be obliged to deny yourselves many things for a time, but, once started, it will be easy, and if you owe no one you will never have it said of you, as it is of the majority of the profession, 'Let him wait, he always makes others wait.'

"Do you know that physicians as a class, are considered by business men among their poorest paying customers? How often you hear it said of Dr. A. or B.: 'He is poor pay, but then he never asks anyone for his pay'? Why not? Is not a doctor's bill just as fairly earned as a grocer's or a liveryman's? Shame on all this twaddle about the purely professional part of our calling. Is not bread and butter just as necessary to our families as to the lawyer's or merchant's? Are we the only humane and charitable profession on the globe?

"Let us drop a little of our senseless quarreling over 'ethics' and make ourselves a little less the laughing-stock of the rest of mankind. Let us pay our own debts and collect our bills just as business men do, and, above all, let us get down from our highly ethical, purely professional view of mundane affairs and grasp the situation like the merchant, farmer or manufacturer.

"We are none of us above accepting our fees when offered. Is it any worse to ask for them within a reasonable time in a business-like manner, not cringing and afraid if we ask Mr. B. for our pay that he will never employ us again? Why should we care to retain his patronage if he don't pay? It isn't all fun to practice medicine. Experience won't pay store bills. The wagon manufacturer won't think of the ethical part of our profession when we need a new buggy to replace the one worn out in the service of the public. The horse-jockey will try just as hard to cheat us, when we need a new horse to take old Jim's place, as though we belonged to the 'honorable' profession of barbers, or tailors or merchants.

"In the many years of my practice I have never sued a man, and I think I can show as small a per centage of poor debts on my books as any physician with the same amount of practice, but I have made it a rule to pay my bills promptly and to call for pay for my own services like other business men. If anyone 'kicks' I tell him plainly but firmly, 'I have a family to support and pay my bills, and to do this I must have pay for my services.' If I find patients are dead-beats, I get rid of them, unless their case is urgent, in which case I always go as a matter of humanity. If a dead-beat undertakes to get into any altercation, I simply say, 'I have never asked you to employ me; probably there are other physicians who will gladly take your case.'

"If I find a patient destitute, I attend to the termination of the case to the best of my ability, and then say: 'I have no bill to make; if you are ever able, you can come and pay me; if not, you are welcome to what I have done.'

"Physicians are largely to blame for their own financial conditions. 'Those who are enabled by law to escape the payment of their debts' are obliged to buy meat, flour and clothing. Are we under greater obligations to them than the merchant? It is not necessary to be inhuman or grasping in order to collect just debts.

"Make fees reasonable and then ask for them like men, with the consciousness of having fully and justly earned them.

"Nine-tenths of all the fault found with physicians is by those who owe them; the patient who pays promptly rarely complains of the doctor.

"Pay as you go, and 'Do unto others as you would be done by' are just as good maxims for a physician to follow as any one else.

"In conclusion, my friends, to use a Scriptural expression (I believe), Life is too short to collect slow debts!"

